# Evaluating the diagnostic performance of MRI-based signs for identification of meniscus posterior root tears: a systematic review and meta-analysis

**DOI:** 10.1186/s13018-025-06592-4

**Published:** 2026-01-08

**Authors:** Chenyang Meng, Tiexin Zhang, Qi Cheng, Changxu Han, Xiao Ma

**Affiliations:** 1 Sports Medicine Center, The Second Affiliated Hospital of Inner Mongolia Medical University, Hohhot, Inner Mongolia China; 2 Graduate School, Inner Mongolia Medical University, Hohhot, Inner Mongolia China; 3 Orthopedics Department, The 969th Hospital of the Joint Logistic Support Force of P.L.A,, Hohhot, Inner Mongolia China

**Keywords:** Meniscus posterior root, Tear, Magnetic resonance imaging, Diagnosis

## Abstract

**Purpose:**

To investigate the diagnostic performance of magnetic resonance imaging (MRI)-based signs for meniscus posterior root tears (MPRTs).

**Materials and methods:**

A literature search was performed to identify original studies published prior to May 15, 2025 that evaluated the diagnostic accuracy of MRI-based signs for MPRTs. All meta-analyses were performed using Meta-DiSc 1.4 software and StataMP 18.

**Results:**

This meta-analysis evaluated 9 MRI-based signs across 8 studies (1430 patients, 1,533 MRI examinations). In terms of pooled sensitivity, the cleft sign and ghost sign were ≥  0.8, while the radial tear sign was only 0.6. The cleft sign and/or truncated triangle sign showed the highest sensitivity (0.91). In terms of pooled specificity, the cleft sign and ghost sign were ≥ 0.85. The radial tear sign showed the highest specificity (0.97). In terms of pooled positive likelihood ratio (PLR), the radial tear sign was the highest (18.56). The cleft sign and ghost sign were ≥  10. In terms of pooled negative likelihood ratio (NLR), the cleft sign and/or truncated triangle sign was the lowest (0.13). The cleft sign and ghost sign were ≤ 0.21, while the radial tear sign was 0.41. In the area under the summary receiver operating characteristic curve (AUC), the ghost sign was the highest (0.97). The cleft sign and radial tear sign were ≥  0.9. In the diagnostic odds ratio (DOR), the cleft sign was the highest (85.32). The ghost sign, radial tear sign and the cleft sign and/or truncated triangle sign were all ≥  50. Subgroup analyses revealed no statistically significant differences (*P* > 0.05).

**Conclusion:**

The cleft sign demonstrated the best overall diagnostic performance. High-sensitivity signs are recommended for initial screening, whereas high-specificity signs are indicated for diagnostic confirmation. The ghost sign and cleft sign, with their high AUC and DOR, serve as core diagnostic criteria.

**Supplementary Information:**

The online version contains supplementary material available at 10.1186/s13018-025-06592-4.

## Introduction

The meniscus posterior root tear (MPRT) is defined as a tear within 1 cm of the root attachment or an avulsion at the root attachment [1, 2]. The fibers of the meniscus root are radially arranged, playing a critical role in load transmission [3, 4]. Root tears account for 10–21% of all meniscus tears, with an estimated incidence of 60–70 cases per 100,000 individuals. MPRTs are the most common subtype [1, 4, 5].

**Fig. 1:**
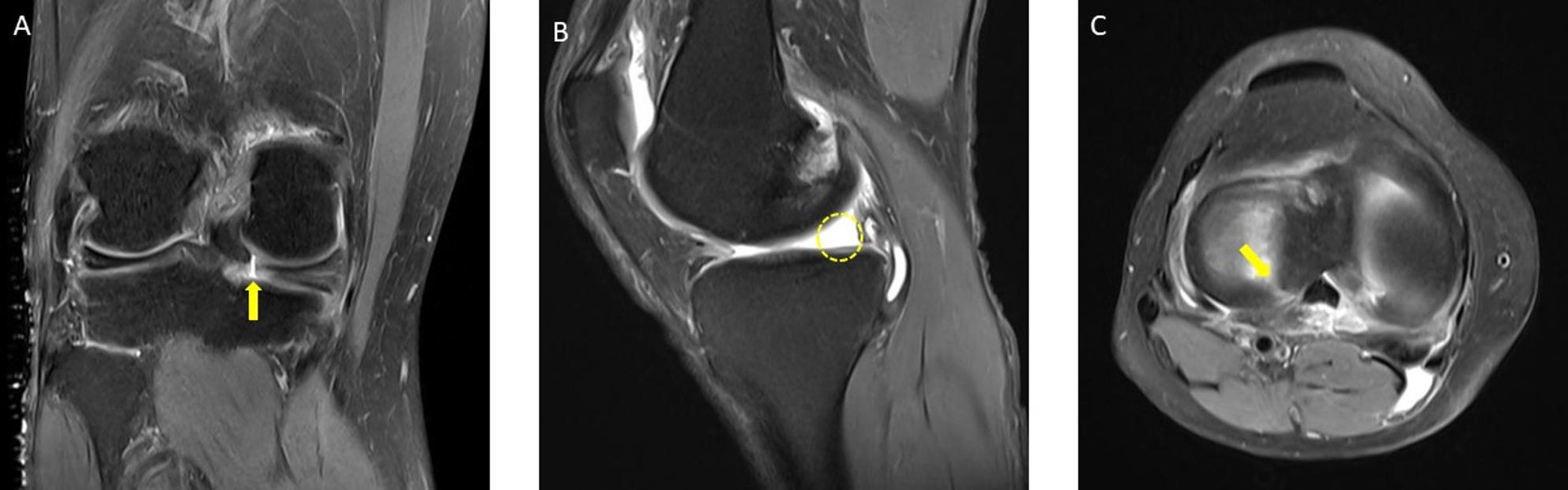
Representative images of magnetic resonance imaging (MRI) signs for meniscus posterior root tears (MPRTs).** A**, the cleft sign (indicated with an arrowhead, coronal image);** B**, the ghost sign (surrounded by a circle, sagittal image);** C**, the radial tear sign (indicated with an arrow, axial image)

**Fig. 2:**
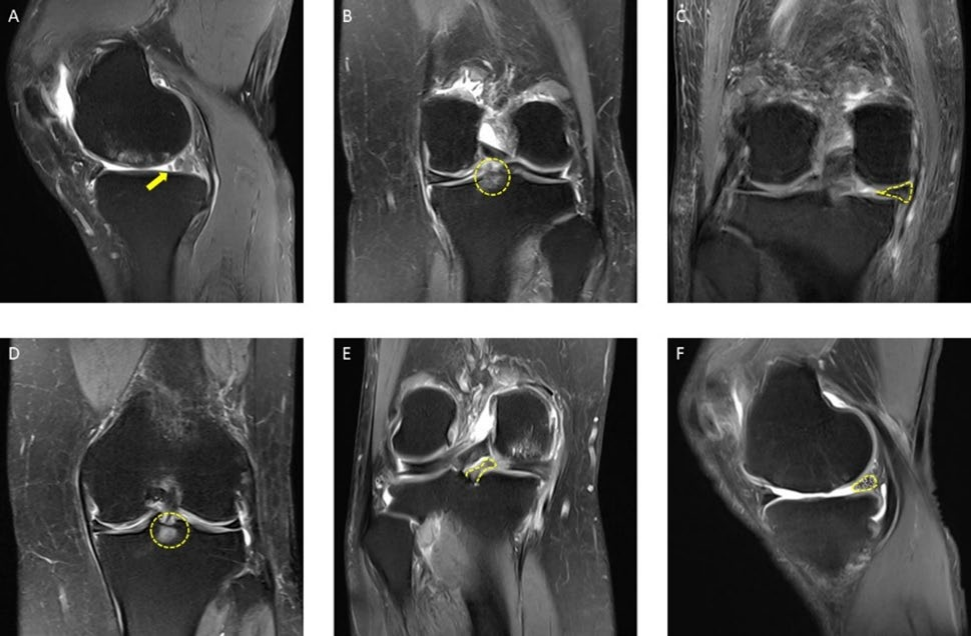
Representative images of magnetic resonance imaging (MRI) signs for meniscus posterior root tears (MPRTs).** A**, The truncated triangle sign (indicated with an arrowhead, sagittal image);** B**, the posterior shiny corner lesion (PSCL) on coronal images (surrounded by a circle, coronal image);** C**, the giraffe neck sign (dotted area, coronal image);** D**, the bone marrow spot under the posterior root attachment (dotted circle, coronal image);** E**, the root irregularity sign (dotted area, coronal image);** F**, the ocarina sign (dotted area, sagittal image)

Physical examination exhibits low sensitivity and specificity for diagnosing MPRTs. Although arthroscopy is the gold standard, its invasive nature and high cost limit its routine use [4]. Thus, magnetic resonance imaging (MRI), with its high soft-tissue contrast and non-invasive approach, has become the primary diagnostic tool for preoperative planning [6]. Certain MRI findings, such as the cleft sign, the ghost sign, and the radial tear sign, have been associated with MPRTs [7–13] (Figs. 1, 2). While current research highlights the diagnostic potential of these MRI-based signs, challenges remain in their application [5, 10–13].

Previous studies reported variable diagnostic performance for these MRI-based signs. For example, Yoshihara et al. [8] found the ghost sign had low sensitivity (0.14) and a low AUC (0.57). Whereas Some studies reported moderate sensitivity for the cleft sign, the ghost sign, and the truncated triangle sign [7, 8, 14, 15]. Conversely, Furumatsu et al. [8, 11] demonstrated that combining multiple signs (e.g., the giraffe neck sign, the cleft sign, the ghost sign, and the radial tear sign) improved diagnostic performance. Given these inconsistencies, a quantitative synthesis of MRI-based signs is warranted.

Therefore, our study aimed to evaluate the diagnostic performance of individual MRI-based signs for MPRTs. We hypothesized that our findings can provide a reliable evidence-based basis for clinicians to reduce missed diagnoses (via high-sensitivity screening) and false positives (via high-specificity confirmation).

## Methods

This study utilized the Preferred Reporting Items for Systematic Reviews and Meta-Analyses for Diagnostic Test Accuracy (PRISMA-DTA) checklist [16]. The protocol of this review has been registered on PROSPERO (ID: CRD420251042114).

### Search strategy

Two independent investigators systematically searched PubMed, Embase, and the Cochrane Library for relevant articles from inception until May 15, 2025. The search strategy combined MeSH terms and free-text keywords ((((meniscus) OR (menisci)) AND (root)) AND ((Magnetic Resonance Imaging) OR (MR))). Any discrepancies between the two investigators were resolved through discussion with a third reviewer.

## Eligibility criteria

Eligible studies were English-language publications investigating the association between MRI-based signs and MPRTs. Exclusion criteria comprised: (1) case reports, (2) reviews articles, (3) cadaver studies, (4) animal studies, (5) consensus statements, (6) editorials/letters, and (7) studies lacking data for a 2  ×  2 table. The two authors independently screened all articles, with inter-rater agreement assessed using Cohen’s kappa (κ) coefficients.

## Quality assessment

The two authors independently assessed the quality of each included study using the Quality Assessment of Diagnostic Accuracy Studies-2 (QUADAS-2) tool [17], evaluating the risk of bias and applicability concerns. This checklist comprises 4 main domains: patient selection, index test, reference standard, and flow and timing. For each study, risk of bias was evaluated in all four domains, while applicability concerns were assessed for the first three domains. The tool enabled the reviewer to rate the risk of bias in each domain as high, low, or unclear. Disagreements between the two reviewers were also resolved by the third reviewer.

## Data extraction and meta-analysis

The two authors independently extracted data on true positives, false positives, false negatives, and true negatives using standardized forms. To address zero-cell entries, a continuity correction of 0.5 was applied to all empty cells in the 2  ×  2 contingency tables.

All statistical analyses were performed using Meta-DiSc (version 1.4; Ramon y Cajal Hospital) software and StataMP 18. Pooled estimates of diagnostic performance measures, including the sensitivity, specificity, diagnostic odds ratio (DOR), positive likelihood ratio (PLR), negative likelihood ratio (NLR), and the area under the summary receiver operating characteristic (SROC) curve (AUC), were estimated and all estimates were reported with their corresponding 95% confidence intervals (CIs). Heterogeneity was evaluated using the Cochrane *Q* test (*P* < 0 .05 indicates significant heterogeneity) and *I*^*2*^ (*I*^*2*^  ≥  50% indicates substantial heterogeneity). For studies demonstrating significant heterogeneity, we employed the DerSimonian-Laird random effects model. To explore potential sources of heterogeneity, we conducted sensitivity analyses using meta-regression. And publication bias was assessed using Deeks’ funnel plot asymmetry test. For certain MRI-based signs where quantitative synthesis was not feasible, we performed descriptive comparisons of the raw data.

To ensure the statistical independence of data points, a sensitivity analysis was conducted based on the principle of “one dataset per MRI sign per independent cohort.” For any primary study that reported multiple stratified datasets (e.g., by timepoint) for the same MRI sign, these datasets were consolidated into a single representative dataset prior to inclusion in the meta-analysis. This sensitivity analysis was performed to confirm that the potential non-independence of such repeated measurements did not bias the pooled estimates.

## Definition of MRI signs

- The cleft sign / the truncation sign: a vertical linear defect on coronal images [7–9, 12, 15, 18, 19].
- The ghost sign / the white meniscus sign: the disappearance of the posterior root with a fluid gap on sagittal images [7–9, 12, 15, 18, 19].
- The radial tear sign: a radial defect on axial images [8, 12, 15, 18].
- The truncated triangle sign: an abrupt termination of the normal triangular meniscus contour on sagittal images [9, 19].
- The posterior shiny corner lesion (PSCL): a focal high signal at the root attachment on T2 fat-suppressed images [8, 20].
- The giraffe neck sign: truncated, swollen posterior segment resembling the long neck of a giraffe on coronal images [7, 18].
- The bone marrow spot: focal high signal in subcortical bone at the root attachment, with rounded and well-defined margins, and no internal bone marrow tissue or Trabecular bone on both T2- and PD-weighted images [7].
- The root irregularity sign: irregular root morphology on coronal images [7, 8].
- The ocarina sign: ocarina-like appearance showing several condensed circles in the posterior horn on sagittal images [7].

## Results

### Literature search

**Fig. 3:**
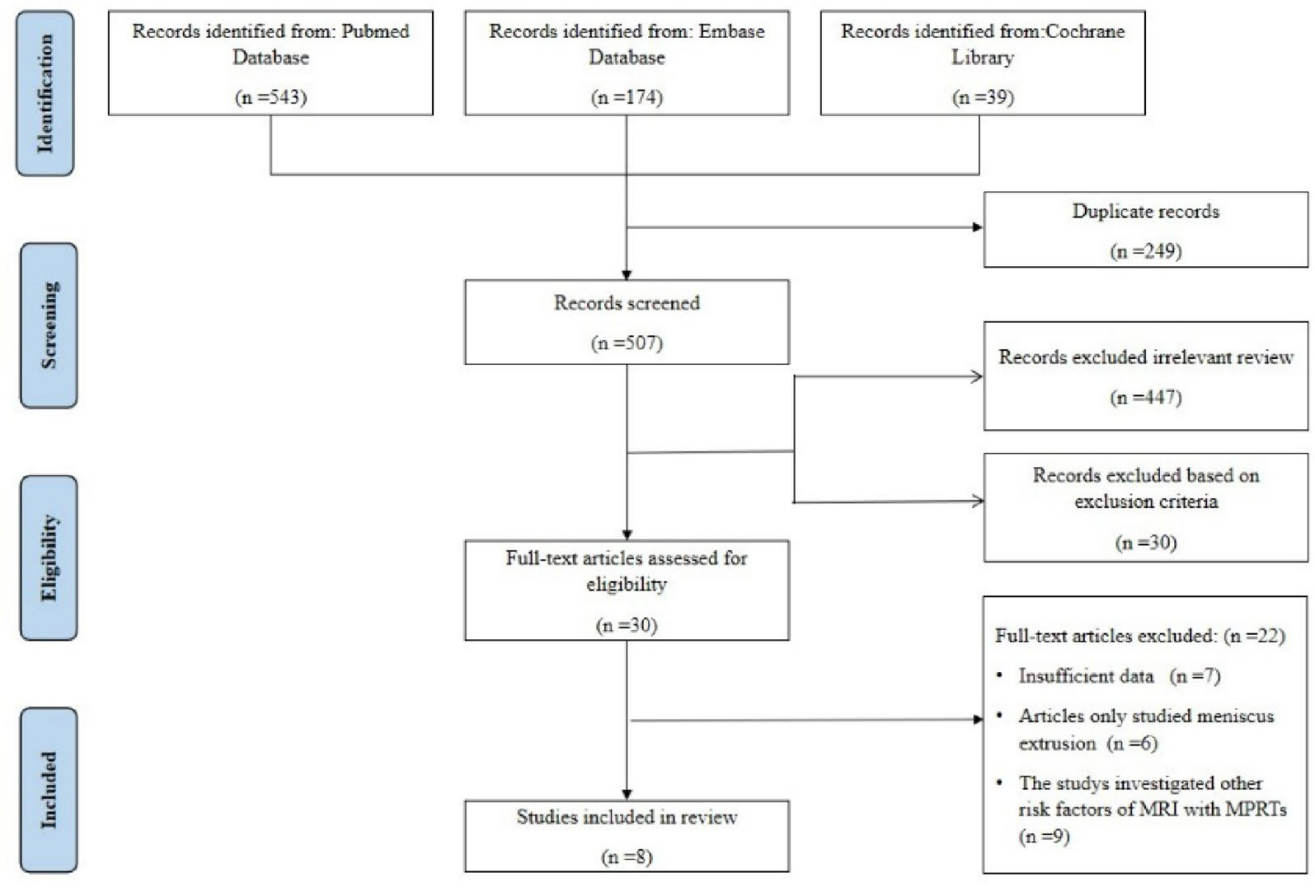
Study selection flowchart according to the preferred reporting items for systematic meta-analyses (PRISMA) statement

Figure 3 presents the PRISMA flow diagram of the systematic review process. Our initial database search identified 756 potentially relevant studies. After removing 249 duplicates using EndNote software (Clarivate Analytics, Philadelphia, PA), we screened 507 unique records. During the title and abstract screening phase, we excluded 447 studies that were not relevant to our research question. An additional 30 studies were excluded based on our predefined exclusion criteria. Following full-text assessment of the remaining 30 studies, we excluded: 7 studies were excluded due to insufficient data, 6 studies were excluded as they only studied meniscus extrusion, and 9 studies were excluded because they investigated other risk factors of MRI finds with MPRTs. A total of 8 studies [7–13, 21] were selected for the final meta-analysis.

## Characteristics of the studies

Table 1 summarizes the key characteristics of the included studies. The systematic review included 8 studies (all retrospective in design) comprising 1430 patients. These studies collectively analyzed 9 different MRI signs through 1533 separate MRI examinations. The two reviewers were blinded in all studies.

**Table 1:** Characteristics of included studies

Study	No. of MRI	Torn site	Age (yr)	Male (%)	BMI (kg/m2)	κ or ICC	MRI interpreters (field strength)	Arthroscopies observers	Characteristics of patients
Yoshihara A [13]	252	L	> 15	50	NR	NR	Orthopedic surgeons (1.5 or 3.0T)	Orthopedic surgeons	The same cohort of patients with ACL injury but with other lateral meniscus injuries were excluded.
Omae H [21]	550	M	61.3 ± 10.6	18	26.1 ± 7.5	κ > 0.90	NR (1.5T)	NR	Patients who underwent arthroscopic surgery and without a memory of onset (painful popping), prior knee surgeries, concomitant ligament or meniscus injuries or fractures were excluded.
Kaneko S [12]	184	M	54.4 ± 16.0	18	25.1 ± 4.7	κ > 0.75	Orthopedic surgeons (NR)	No arthroscopy	Outpatients with painful popping but an unclear date of injury and patients without painful popping were excluded.
Furumatsu T [11]	46	M	59.3 ± 8.7	11	25.7 ± 3.8	ICC > 0.80	Orthopedic surgeons (1.5T)	NR	Patients confirmed partial tears of the medial meniscus posterior root and other types of medial meniscus tears.
Nguyen JC [10]	90	L	15.5 ± 1.6	5	23.7 ± 4.4	κ > 0.80	Radiologists (1.5 or 3.0T)	Orthopedic surgeons	Children who underwent MRI within 90 days before arthroscopic primary ACL reconstruction.
Asai K [9]	231	L	20.3 ± 14.8	56	22.3 ± 3.5	κ > 0.80	Orthopedic surgeons (1.5T)	Orthopedic surgeons	Patients who underwent primary ACL reconstruction.
Furumatsu T [14]	120	M	57.8	16	26.2 ± 4.5	κ > 0.80	NR (1.5T)	NR	Patients who underwent surgical treatments for the media MPRT and other types of medial meniscus tears.
Choi SH 2012	60	M	52.0	23	NR	κ > 0.75	Radiologists (3.0T)	NR	Patients who were arthroscopically confirmed as having medial meniscal root tear.

MRI, magnetic resonance imaging; M, posterior root of the medial meniscus; L, posterior root of the lateral meniscus; MPRTs, meniscus posterior root tears; yr, year; Male%, percentage of males; NR, not reported; BMI, body mass index; *κ*, Cohen’s kappa coefficients; ICC, intraclass correlation coefficient; ACL, anterior cruciate ligament; MPRT, meniscus posterior root. Age and BMI are presented as mean ±  standard deviation unless otherwise specified

All 8 included studies evaluated both the cleft sign and the ghost sign, yielding 9 comparative datasets (Kaneko et al. [12] provided two distinct datasets stratified by time since injury: < 1 month and > 1 month, to account for potential signal evolution post-injury). 5 studies [7, 8, 10–12] evaluated the radial tear sign with 6 comparisons. 3 studies [8, 11, 21] evaluated the giraffe neck sign with 3 comparisons. 2 studies [12, 21] evaluated PSCL with 3 comparisons. 1 study [11] evaluated the bone marrow spot sign, providing 2 distinct comparisons by analyzing coronal and sagittal planes separately. 2 studies [9, 13] evaluated the truncated triangle sign with 2 comparisons. 1 study [11] evaluated both the ocarina sign and the root irregularity sign, providing 1 comparative dataset for each signs. Among combinations of MRI signs, the cleft sign and/or truncated triangle sign was assessed in 2 studies [9, 13] with 2 comparisons. All other combinations were reported in single studies with 1 comparison. Datasets with ≥ 2 comparisons were combined and those with ≥ 3 comparisons underwent SROC analysis with AUC calculation. No adjustments were made for incidence rates in the included studies. The majority of studies had consistency coefficients above 0.75, with five studies exceeding 0.8 and one study [13] not reporting this measure.

### Quality assessment

**Fig. 4:**
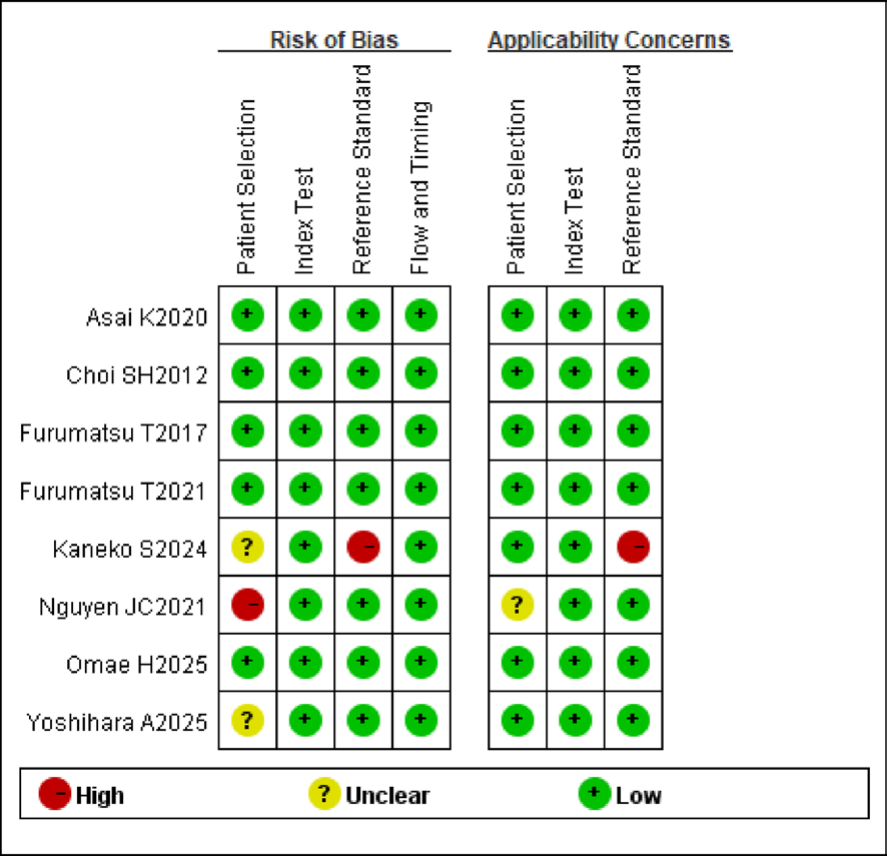
The quality assessment of diagnostic accuracy studies-2 (QUADAS-2) domains for the included studies

The methodological quality of included studies, assessed using the QUADAS-2 tool, is summarized in Fig. 4. Most studies demonstrated low risk of bias across all domains. However, three studies warranted specific concerns. One study [12] had a high risk of bias with respect to the reference standard and the applicability domains and a unclear risk of bias with respect to patient selection, as the study excluded patients with an unclear date of injury and was not determined using arthroscopy. One study [10] had high risk of bias with respect to patient selection and unclear risk of bias with applicability domains, because of pediatric-only cohort. One study [13] had unclear risk of bias with respect to patient selection because of high heterogeneity of included patients and unclear exclusion criteria. All other studies showed low risk in all domains.

### Diagnostic performance

**Fig. 5:**
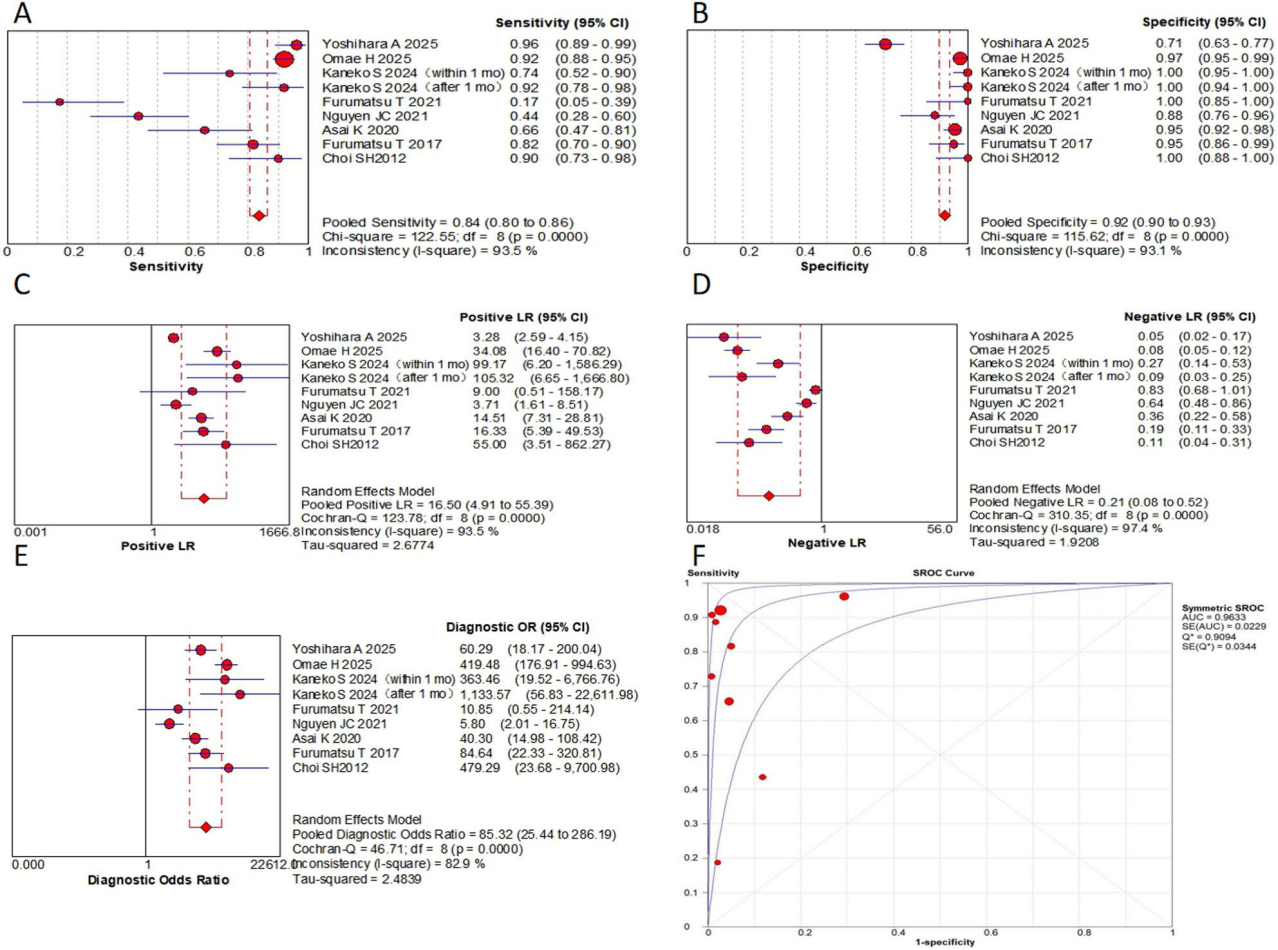
**A** to** E** are forest plots (**A**, the pooled sensitivity,** B**, the pooled specificity,** C**, the PLR;** D**, the NLR;** E**, the DOR) of the cleft sign;** F** are SROC for the cleft sign

**Fig. 6:**
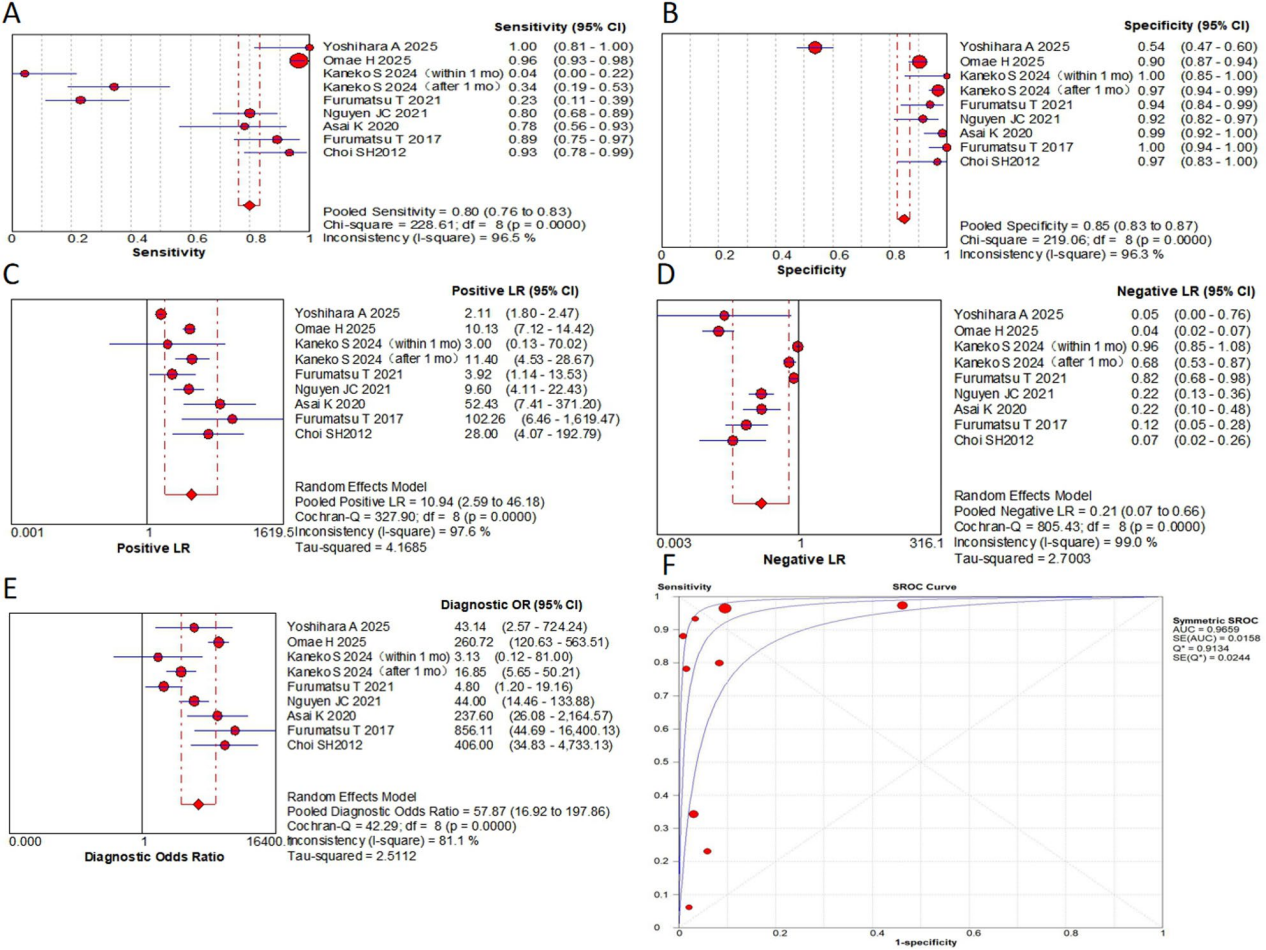
**A** to** E** are forest plots (**A**, the pooled sensitivity, ** B**, the pooled specificity, ** C**, the PLR; ** D**, the NLR; ** E**, the DOR) of the ghost sign; ** F** are SROC for the ghost sign

**Fig. 7:**
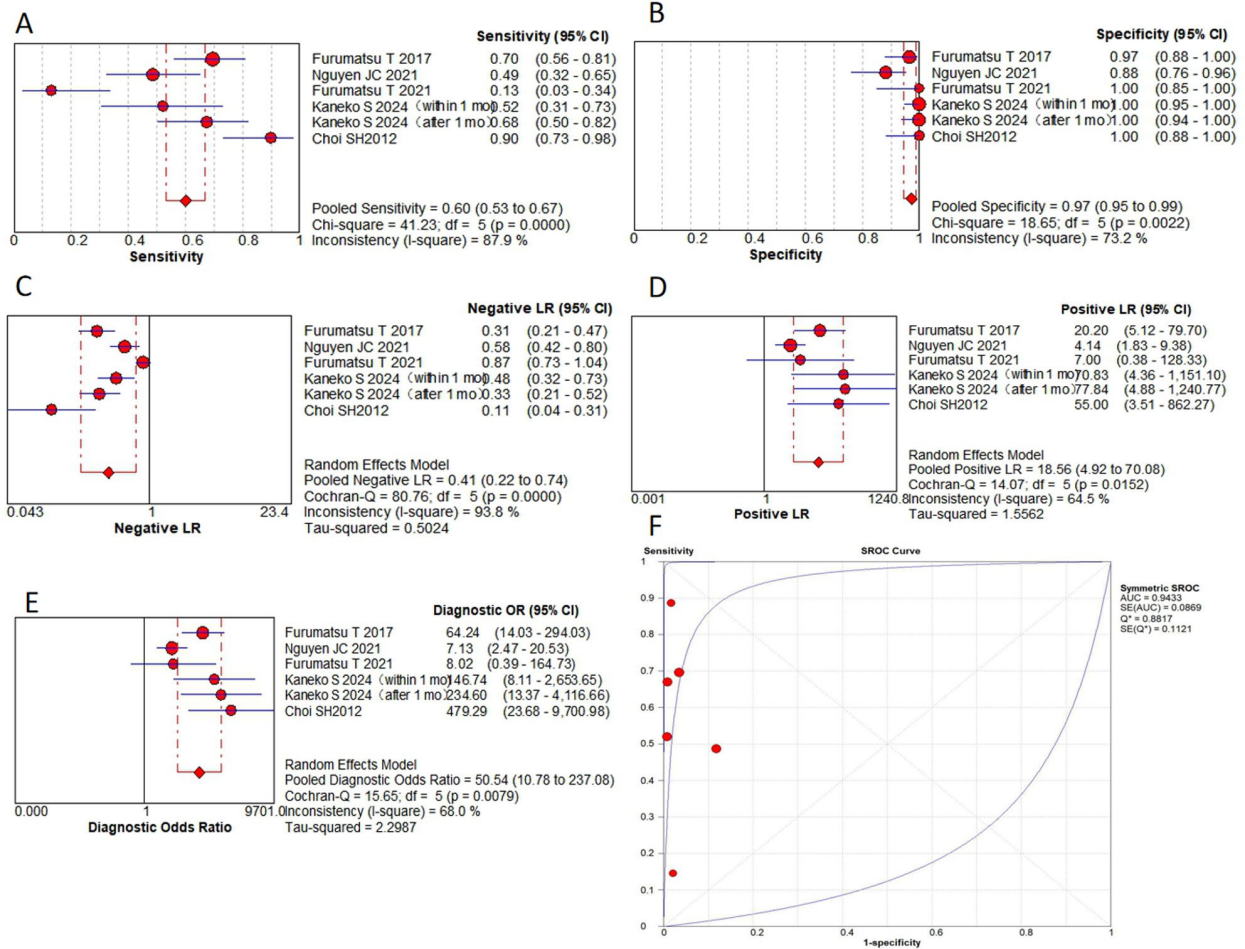
**A** to** E** are forest plots of forest plots (**A**, the pooled sensitivity, ** B**, the pooled specificity, ** C**, the PLR; ** D**, the NLR; ** E**, the DOR) of the radial tear sign; ** F** are SROC for the radial tear sign

This meta-analysis evaluated 9 MRI-based signs from 8 original research articles, containing 1430 patients with a total of 1,533 MRI examinations. Key findings include: in terms of pooled sensitivity, the truncated triangle sign, the cleft sign, the ghost sign and the giraffe neck sign all ≥  0.8, while the radial tear sign was only 0.6. The cleft sign and/or truncated triangle sign showed the highest sensitivity (0.91). In terms of pooled specificity, the cleft sign and ghost sign were 0.92 and 0.85, respectively. The radial tear sign showed the highest specificity (0.97). The bone marrow spot and the cleft sign and/or truncated triangle sign were also ≥  0.85. In terms of pooled PLR, the radial tear sign was the highest (18.56). the cleft sign and the ghost sign were ≥  10. In terms of pooled NLR, the cleft sign and/or truncated triangle sign was the lowest (0.13). The truncated triangle sign, the cleft sign and the ghost sign were all ≤  0.21, while the radial tear sign was 0.41. In the AUC, the ghost sign was the highest (0.97). The cleft sign, the radial tear sign and the giraffe neck sign were all ≥ 0.9. In the DOR, the cleft sign was the highest (85.32). the ghost sign, the radial tear sign and the cleft sign and/or truncated triangle sign were all ≥  50. Subgroup analyses revealed no statistically significant differences (*P* >  0.05). Complete results are presented in Figs. 5, 6 and 7; Table 2.

**Table 2:** Results of the pooled analysis

	Sensitivity	95% CI	Specificity	95% CI	PLR	95% CI	NLR	95% CI	AUC	Q	DOR	95% CI
Ghost sign	0.80	0.76–0.83	0.85	0.83–0.87	10.94	2.59–46.18	0.21	0.07–0.66	0.97	0.91	57.87	16.92-197.86
Cleft sign	0.84	0.80–0.86	0.92	0.90–0.93	16.5	4.91–55.39	0.21	0.08–0.52	0.96	0.91	85.32	25.44-286.19
Radial tear sign	0.60	0.53–0.67	0.97	0.95–0.99	18.56	4.92–70.08	0.41	0.22–0.74	0.94	0.88	50.54	10.78-237.08
Giraffe neck sign	0.80	0.73–0.85	0.65	0.61–0.69	7.97	0.52-123.04	0.27	0.04-2.00	0.92	0.85	33.04	7.47-146.16
Posterior shiny corner lesion	0.73	0.66–0.79	0.68	0.64–0.72	4.43	1.19–16.46	0.31	0.03–3.55	0.88	0.81	14.59	3.62–58.83
Truncated triangle sign	0.84	0.76–0.90	0.84	0.79–0.87	5.45	1.84–16.13	0.21	0.04–1.05			27.32	14.4-51.81
Cleft sign and/or Truncated triangle sign	0.91	0.84–0.95	0.88	0.84–0.91	6.88	3.63–13.02	0.13	0.04–0.45			55.71	28.43-109.17
Bone marrow spot	0.41	0.27–0.57	0.89	0.76–0.96	3.79	1.55–9.26	0.67	0.52–0.87			5.89	1.95–17.77

DOR, diagnostic odds ratio; PLR, positive likelihood ratio; NLR, negative likelihood ratio; AUC, the area under the summary receiver operating characteristic curve; *Q*, cochrane *Q* test; 95% CI, corresponding 95% confidence interval

A sensitivity analysis was performed to address potential non-independence arising from multiple datasets for the same MRI sign within a study. For each affected sign, a single dataset was selected from the available ones for inclusion, and the meta-analysis was rerun. The recalculated pooled estimates for the cleft sign (sensitivity: 0.84, specificity: 0.91, AUC: 0.96, DOR: 75.27), ghost sign (sensitivity: 0.83, specificity: 0.85, AUC: 0.97, DOR: 72.91) and radial tear sign (sensitivity: 0.61, specificity: 0.96, AUC: 0.96, DOR: 43.11) were not substantially different from the primary analysis, confirming the robustness of our results.

### Meta-regression analyses

**Fig. 8:**
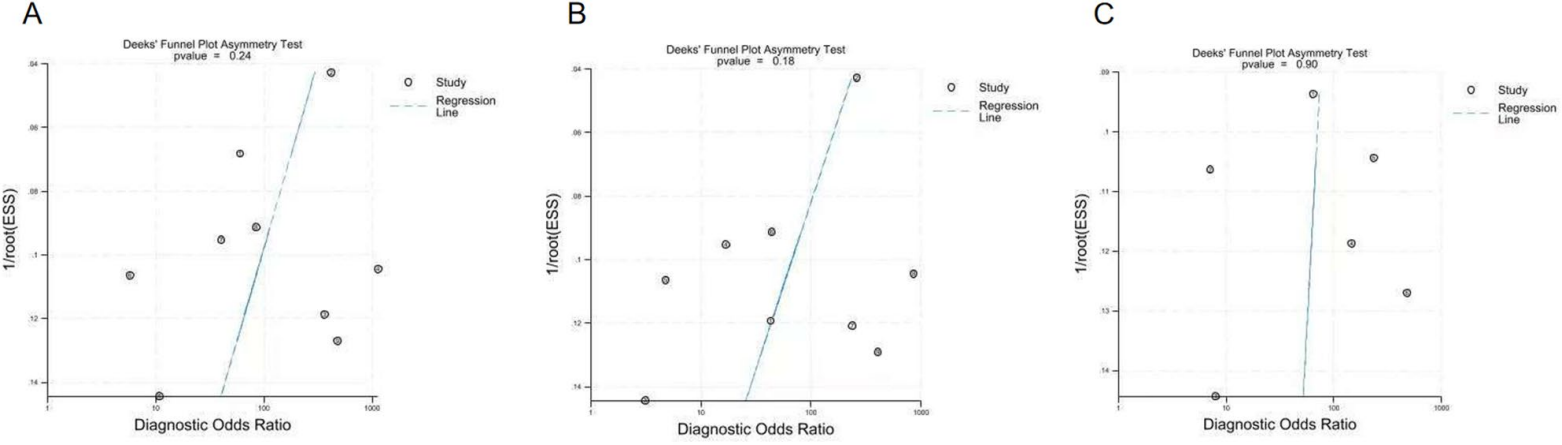
Funnel plots (**A**–**C**) from Deeks’ asymmetry test for publication bias. The plots correspond to the diagnostic accuracy meta-analyses for the cleft sign (**A**), ghost sign (**B**), and radial tear sign (**C**)

Table 3 presents the findings of our meta-regression analysis, which examined potential sources of heterogeneity across five predefined subgroups: torn site (medial vs. lateral MPRTs), concomitant injury (presence vs. absence of ACL injury), reference standard (with vs. without of arthroscopy as gold standard), age stratification (mean age  ≥ 50 vs. <  50 years) and study size: ≥ 100 versus <  100 MRI examinations per dataset. The analysis revealed no statistically significant differences in diagnostic performance across any of these subgroup comparisons (*P* > 0 .05) (Fig. 8).

**Table 3:** Meta-regression analyses regarding possible sources of heterogeneity

	Variable	*P* value	RDOR	95% CI
Cleft sign	Torn site/ACL injuries	0.93	1.34	(0.00–15113.55)
	arthroscopies	0.17	16.21	(0.11–2341.02)
	Mean age	0.57	4.75	(0.00–12264.50)
	No. of MRI	0.46	0.39	(0.01–14.03)
Ghost sign	Torn site/ACL injuries	0.26	4.91	(0.12-194.72)
	arthroscopies	0.90	0.81	(0.00-137.71)
	Mean age	0.56	1.84	(0.09–35.85)
	No. of MRI	0.37	0.26	(0.00–14.79)
Radial tear sign	Torn site/ACL injuries/Mean age	0.24	62.8	(0.00–43364450035.18)
	arthroscopies	0.46	5.79	(0.00–1954408863.58)
	No. of MRI	0.52	4.28	(0.00–1533055764.80)

ACL, anterior cruciate ligament; MRI, magnetic resonance imaging; 95% CI, corresponding 95% confidence interval; RDOR, relative diagnostic odds ratio

## Discussion

A significant finding from this study was that each sign had its own diagnostic advantage in diagnosing MPRTs. The cleft sign exhibited superior overall diagnostic efficacy (DOR of 85.32, AUC of 0.96). The radial tear sign demonstrated the highest specificity (0.97) and PLR (18.56), albeit with limited sensitivity (0.60). The ghost sign, while showing moderately lower specificity (0.85), maintained high diagnostic value (AUC of 0.97) as a supplementary indicator. Combinations of MRI signs can significantly improve the diagnostic accuracy. The DOR of the cleft sign and/or truncated triangle sign was 55.71, and the sensitivity increased to 0.91. Other signs and combinations also showed clinically meaningful diagnostic value. This study may provide valuable insights for clinical diagnostic process. When evaluating a patient, clinicians may consider prioritizing the assessment of the cleft sign. A positive cleft sign could strongly suggest the presence of MPRTs, warranting further investigation. Conversely, if the cleft sign is negative, the radial tear sign should be evaluated as an additional diagnostic marker. The ghost sign may serve as a supplementary indicator to support the overall diagnostic assessment.

Some studies have found that the ocarina sign was most prevalent in partial MPRTs [11]. Some studies have also found that PSCL has a better diagnostic performance in the early stage of MPRTs, and it even outperformed the cleft sign, the ghost sign and the radial tear sign [12, 21–23]. As the disease progresses, PSCL would develop into noble cysts [21]. In this study, we verified that the truncated triangle sign, the giraffe neck sign and PSCL all have high sensitivity ≥ 0.7, indicating that these signs have a low false-negative rates for screening. The specificity of the root irregularity sign, the bone marrow spot, the ocarina sign and the truncated triangle sign was all ≥ 0.8, suggesting that these signs have a reliable for rule-in diagnosis. The PLR of the giraffe neck sign, the root irregularity sign, the truncated triangle sign and the ocarina sign was also ≥ 5, suggesting that the likelihood of MPRTs was significantly increased when these signs were present. The NLR of the truncated triangle sign and the giraffe neck sign was ≤ 0.3, indicating that the likelihood of MPRTs was low when these signs were not present. The AUC of the giraffe neck sign and PSCL was ≥ 0.85, indicating its high diagnostic accuracy. The DOR of the giraffe neck sign, the truncated triangle sign, the ocarina sign and PSCL was also ≥ 10, suggesting that these signs have a strong combined discriminatory power for the diagnosis of MPRTs.

Combinations of MRI signs occasionally outperformed individual MRI-based signs in diagnostic performance. In the present study, all of combinations of MRI signs showed DOR of ≥ 30, and outperformed any of the individual MRI-based signs in terms of sensitivity and PLR. Such results may be due to the fact that MPRTs patterns are often radiological tears [10, 11, 18, 24], which are more easily detected in axial MR but there are many difficulties in obtaining proper axial MR images. Whereas, when this tear is observed in coronal or sagittal position, if the orientation of the MRI slice is not parallel or perpendicular to the site of rupture, it may not be recognized as a cleft sign or a ghost sign but may be recognized as a truncated triangular sign [13]. The ghost sign is diagnosed only on single sagittal slices perfectly aligned with the rupture site, whereas clefts and truncated triangular signs may be detected on multiple images [9]. This may explain why the cleft sign in the coronal plane, the ghost sign in the sagittal plane, and the radial tear sign in the axial plane show high specificity for diagnosing MPRTs, and why the cleft sign and/or truncated triangle sign is more accurate in such diagnoses.

In our study, ACL injury and medial LPRT were always concomitant, which may be related to the increased stress on medial LPRT due to altered knee biomechanics after ACL injuries. In addition, key limiting factors for lateral root assessment: the presence of pulsation artifacts in the popliteal artery, volume averaging of small structures, and the magic angle effect (due to the tilting of the medial meniscus over the tibial tuberosity convexity), as well as complex anatomical structures related to the origins of the ligament of the femur [1, 19], all of which contribute to the higher detection rates of MRI for diagnosing medial MPRTs than lateral MPRTs.

MPRTs are frequently associated with a number of additional imaging manifestations such as, medial femoral ligament deficiency, meniscotibial ligament tears, lateral femoral notch sign, bone cysts at the posterior root attachment, regional synovitis, medial femoral-tibial compartment osteophyte, meniscus extrusion, lateral joint space widening, flattening of the lateral femoral condyle, depression of the lateral tibial plateau, hypoplasia of the lateral tibial spine, elevation of the fibular head and condylar cutoff sign. These lesions have been associated with femoral prominence and subchondral insufficiency fractures [25–33]. In addition, the presence of subchondral fractures significantly correlates with the width of the gap of the root tear and the extent of the root lesion [33, 34]. It should be noted here that this review did not statistically analyze the data related to the signs of meniscal extrusion, as several studies [24, 35–40] have clearly and comprehensively explored it. For example, a systematic review and meta-analysis by Daniel et al. [40] provided specific scientific techniques for measuring meniscal bulging and bulging values. Another systematic review and meta-analysis by Meng et al. [41] provided a detailed summary of 17 studies validating the correlation between lateral meniscal extrusion and MPRTs.

### Limitations

Several limitations need to be elaborated. First, this study was designed to include more MRI-based signs and to perform quantitative analyses and included a study without a gold standard, which led to a greater mix of articles that we included, and the validity of sensitivity analyses may have been compromised. Secondly, the limited number of included studies resulted in underpowered subgroup and meta-regression analyses; furthermore, for several signs and combinations of MRI signs (e.g., the ocarina sign, the bone marrow spot), the available data were too scarce to even permit a meta-analysis. In addition, the evidence synthesized in this review is subject to both demographic bias, as most studies involved East Asian populations, and selection bias, since all included studies were retrospective in design and the most MRI interpretations were conducted by orthopedic surgeons rather than radiologists. Finally, some primary studies contributed data for multiple MRI signs from the same patient cohort. While our primary analysis treated these as independent, this may theoretically violate the assumption of statistical independence. However, a pre-specified sensitivity analysis that corrected for this potential bias demonstrated that our primary results and conclusions remained robust. However, we also ran the correlation analysis again, and the results were relatively stable after temporarily excluding some of the more extreme results.

The future of MPRTs diagnosis lies in a paradigm shift from quantifying isolated signs to developing integrated and dynamic diagnostic systems. We propose two key research directions to achieve this: first, the development of machine learning classifiers (e.g., Random Forests, Support Vector Machines) that integrate the MRI-based signs identified in this review with relevant clinical variables to generate a unified, high-accuracy diagnostic probability. Second, a critical elucidation of the optimal diagnostic timing is required, specifically how the sensitivity and specificity of key MRI-based signs evolve relative to the time of initial injury. Ultimately, these avenues can converge within a Bayesian probabilistic framework. By incorporating time-sensitive likelihood ratios, such a model could provide clinicians with a continuously updated, patient-specific probability of MPRTs, thereby directly supporting and enhancing the clinical decision-making process.

## Conclusions

MRI-based signs exhibit excellent diagnostic performance for MPRTs, with the cleft sign demonstrating the optimal overall diagnostic efficacy, and the combination of multiple signs can improve the diagnostic accuracy. The high-sensitivity signs (particularly the cleft sign and/or truncated triangle sign or the cleft sign) should be employed for initial screening, followed by confirmation using high-specificity signs (the radial tear sign or the cleft sign and/or ghost sign), while reserving other MRI-based signs and their combinations for adjunctive diagnostic support.

## Supplementary Information

Below is the link to the electronic supplementary material.

Supplementary Material 1.

## Data Availability

The original contributions presented in the study are included in the article/supplementary material, further inquiries can be directed to the corresponding authors.
