# Supplementary material for: Evaluating the diagnostic performance of MRI-based signs for identification of meniscus posterior root tears: a systematic review and meta-analysis
Source: J Orthop Surg Res. 2026 Jan 8;21:94. doi: 10.1186/s13018-025-06592-4 (PMC12870275; doi:10.1186/s13018-025-06592-4)
Supplement: Supplementary file 1 — Supplementary Material 1. [file 13018_2025_6592_MOESM1_ESM.docx]

Table IV. Results of the Heterogeneity Test.

|  | *Q* of DOR | *P* (0.1) | Tau² of DOR | *I²* of DOR | *I²* of sensitivity | *I²* of specificity | *I²* of PLR | *I²* of NLR |
| --- | --- | --- | --- | --- | --- | --- | --- | --- |
| Cleft sign | 46.70 | 0.000 | 2.4839 | 82.90% | 93.50% | 93.10% | 93.50% | 97.40% |
| Ghost sign | 42.29 | 0.000 | 2.5112 | 81.10% | 96.50% | 96.30% | 97.60% | 99.00% |
| Radial tear sign | 15.65 | 0.008 | 2.2987 | 68.00% | 87.90% | 73.20% | 64.50% | 93.80% |

Table V. Results of the Decks' Funnel Plot Asymmetry Test

|  | Coefficient | Std. err. | *t* | *P*>\|*t\|* | [95% conf. interval] |
| --- | --- | --- | --- | --- | --- |
| Cleft sign | -19.6728 | 15.35317 | -1.28 | 0.241 | -55.97731-16.63164 |
| Ghost sign | -21.8129 | 14.75237 | -1.48 | 0.183 | -56.69675-13.07088 |
| Radial tear sign | -6.81235 | 48.81694 | -0.14 | 0.896 | -142.3499-128.7252 |

Table VI. Sensitivity Analysis Based on the "One Dataset Per Independent Cohort" Principle

|  | Cleft sign | | Ghost sign | | Radial tear sign | |
| --- | --- | --- | --- | --- | --- | --- |
|  | Original values | Recalculated values | Original values | Recalculated values | Original values | Recalculated values |
| Sensitivity | 0.84 | 0.84 | 0.80 | 0.83 | 0.60 | 0.61 |
| 95% CI | 0.80-0.86 | 0.81-0.87 | 0.76-0.83 | 0.80-0.86 | 0.53-0.67 | 0.54-0.68 |
| Specificity | 0.92 | 0.91 | 0.85 | 0.85 | 0.97 | 0.96 |
| 95% CI | 0.90-0.93 | 0.89-0.93 | 0.83-0.87 | 0.82-0.87 | 0.95-0.99 | 0.93-0.98 |
| PLR | 16.50 | 13.99 | 10.94 | 12.26 | 18.56 | 15.15 |
| 95% CI | 4.91-55.39 | 4.06-48.21 | 2.59-46.18 | 2.70-192.79 | 4.92-70.08 | 3.72-61.74 |
| NLR | 0.21 | 0.20 | 0.21 | 0.18 | 0.41 | 0.39 |
| 95% CI | 0.08-0.52 | 0.07-0.56 | 0.07-0.66 | 0.06-0.55 | 0.22-0.74 | 0.18-0.81 |
| AUC | 0.96 | 0.96 | 0.97 | 0.97 | 0.94 | 0.96 |
| *Q* | 0.91 | 0.91 | 0.91 | 0.91 | 0.88 | 0.91 |
| DOR | 85.32 | 75.27 | 57.87 | 72.91 | 50.54 | 43.11 |
| 95% CI | 25.44-286.19 | 20.99-269.83 | 16.92-197.86 | 20.71-256.61 | 10.78-237.08 | 7.77-239.15 |
| *Q* of DOR | 46.70 | 45.5 | 42.29 | 38.88 | 15.65 | 14.10 |
| *P* (0.1) | 0.000 | 0.000 | 0.000 | 0.000 | 0.008 | 0.007 |
| Tau² of DOR | 2.48 | 2.56 | 2.51 | 2.41 | 2.30 | 2.50 |
| *I²* of DOR | 0.83 | 0.85 | 0.81 | 0.82 | 0.68 | 0.72 |
| *I²* of sensitivity | 0.93 | 0.94 | 0.97 | 0.96 | 0.88 | 0.90 |
| *I²* of specificity | 0.93 | 0.93 | 0.96 | 0.97 | 0.73 | 0.72 |
| *I²* of PLR | 0.94 | 0.94 | 0.98 | 0.98 | 0.65 | 0.66 |
| *I²* of NLR | 0.97 | 0.98 | 0.99 | 0.98 | 0.94 | 0.95 |
